# Mammalian sperm nuclear organization: resiliencies and vulnerabilities

**DOI:** 10.1186/s12610-016-0044-5

**Published:** 2016-12-21

**Authors:** A. Champroux, J. Torres-Carreira, P. Gharagozloo, J. R. Drevet, A. Kocer

**Affiliations:** 1 GReD “Genetics, Reproduction & Development” Laboratory, UMR CNRS 6293, INSERM U1103, Clermont Université, BP60026 - TSA60026, 63178 Aubière cedex, France; 2 Centro Universitário Rio Preto, UNIRP, Rodovia Br153, Km 69, CEP15093-450 São José do Rio Preto, São Paulo Brazil; 3 CellOxess LLC, 830 Bear Tavern Road, Ewing, NJ 08628 USA

**Keywords:** Sperm DNA damage, Oxidative stress, Infertility, Developmental impacts, Dommage à l’AND spermatique, Stress oxydant, Infertilité, Impacts developmentaux

## Abstract

Sperm cells are remarkably complex and highly specialized compared to somatic cells. Their function is to deliver to the oocyte the paternal genomic blueprint along with a pool of proteins and RNAs so a new generation can begin. Reproductive success, including optimal embryonic development and healthy offspring, greatly depends on the integrity of the sperm chromatin structure. It is now well documented that DNA damage in sperm is linked to reproductive failures both in natural and assisted conception (Assisted Reproductive Technologies [ART]). This manuscript reviews recent important findings concerning - the unusual organization of mammalian sperm chromatin and its impact on reproductive success when modified. This review is focused on sperm chromatin damage and their impact on embryonic development and transgenerational inheritance.

## Résumé

Les spermatozoïdes sont des cellules particulièrement complexes et très spécialisées comparées aux cellules somatiques. Leur rôle est de délivrer dans l’ovule le patrimoine génétique paternel ainsi qu’un lot de protéines et d’ARNs de façon à initier un nouvel individu. Le succès reproductif qui recouvre les aspects de développement embryonnaire harmonieux et de santé de la descendance repose en partie sur l’intégrité de la chromatine spermatique. Les dommages à l’ADN spermatique sont clairement associés aux échecs reproductifs que ce soit en reproduction naturelle et en procréation médicalement assistée (PMA). Cette revue présente les derniers développements concernant l’organisation très particulière de la chromatine spermatique et ses impacts sur le succès reproductif quand cette organisation est perturbée, en particulier sur le développement embryonnaire et les risques trangénérationnels.

## Background

In both female and male, the germline is the only heritable lineage that guarantees the continuity of life. Germ cells are generated during gametogenesis, a sex specific differentiation program taking place in the gonads [1]. During this process, male germ cell maturation is characterized by a massive chromatin remodeling and cellular restructuring. This complex process allows the transformation of diploid spermatogonia into fully cyto-differentiated haploid spermatozoa. Spermatogenesis is highly conserved in mammals and can be subdivided into three major steps: (1) a mitotic amplification step ensuring proliferation and maintenance of spermatogonia, (2) a meiotic step in which spermatogonia evolve into spermatocytes (primary and secondary) ultimately differentiated into spermatids and, finally, (3) a post-meiotic step, also known as spermiogenesis, where spermatids are differentiated into spermatozoa. This last step can be divided into several distinct phases: early spermatids harboring a round nuclei; intermediate spermatids showing an elongated nuclei; and mature spermatozoa with a condensed nuclei [1]. One of the hallmarks of spermiogenesis is the replacement of nuclear somatic-like histones by protamines (small basic proteins) facilitating compaction of the sperm nucleus and, consequently, of the sperm head. In somatic cells the chromatin is organized in nucleosomes containing 146 bp of DNA wrapped around an octamer of histones [2–4]. Chromatin organization and all its associated modifications, whether it concerns the DNA itself and/or the nuclear histones, are critical for gene expression, cell division, and differentiation [5, 6]. In spermatozoa, during meiotic and post-meiotic phases, most of the histones are gradually replaced by testis-specific histone variants followed by the replacement of most histones, first with DNA-interacting non-histones transition nuclear proteins and, subsequently with protamines [7–13]. Sperm DNA-protamine interaction leads to a unique appearance that involves the coiling of sperm DNA into toroidal subunits, also known as “doughnut loops”, containing 50 to 100 kb of DNA [14, 15]. This structure is the consequence of the presence of high level of arginines and cysteines within protamines that mediates strong DNA binding and the formation of inter- and intra-protamine disulfide bonds critical for the optimal compaction of the paternal genomic material. Sperm nuclear compaction is a crucial factor since it is directly related to the sperm head volume and, therefore to the optimal velocity of this cell, a trait that is important for the success of fertilization. In addition, efficient nuclear compaction is critical for the protection of the paternal genomic material against chemical and physical modifications [16]. The main focus of this review concerns the recent advances in the study of sperm chromatin reorganization, sperm chromatin/DNA damage and how they can affect reproductive outcome.

## The male germinal chromatin: a unique and elaborate structure

### The somatic chromatin

At the beginning of spermatogenesis, in spermatogonia and in spermatocytes, the chromatin of germinal cells is identical to that of somatic cells chromatin. It consists of a combination of DNA with small basic nuclear proteins, the histones. These proteins are rich in lysine and arginine residues, giving them a global positive charge allowing their interaction with the negatively charged DNA. This interplay neutralizes a large part of the negative charge of the DNA thus facilitating it to fold in on itself and form compact chromatin that is contained within the tiny nucleus of a cell. The nucleosome, the basic unit of somatic chromatin, as mentioned above is composed of a series of 146 bp of DNA wrapped in 1.67 turns around core histone proteins. This unit is made of an octamer of four histone proteins, H2A, H2B, H3, and H4 (each in two copies), the so-called canonical histones. The full length of the DNA molecule is associated with these nucleosomes and acquires a “beads on a string” structure. A fifth histone, H1, interacts with the DNA sequence bridging nucleosomes and allows for a greater compaction of the chromatin. Histones organize the chromatin into a fiber of 11 nm in diameter, which coils-up upon itself several times into a fiber larger in diameter but shorter in length (for a recent review see: [17]). Each coil corresponds to a new level of organization whose structure is not well understood or controversial because of technical limitations and differences between in vitro (ie. diluted chromatin) and in vivo (ie. concentrated chromatin) conditions [18–21]. The structure of the chromatin is not homogenous and fixed as it seems. The cell nucleus observed *by* transmitted electronic microscopy (TEM) shows areas of variable density depending on the level of chromatin compaction. Clear and less condensed areas in the center of the nucleus correspond to euchromatin, which is more accessible to protein complexes involved in transcription and then contains more active genes. Dark and condensed areas in the nuclear periphery are called heterochromatin that highly represses gene transcription because of its inaccessibility to the transcriptional machinery. Moreover, these areas are variable in function of the cell types and of the level of cellular differentiation.

The transition between euchromatin and heterochromatin is also based on different processes allowing modifications of the physicochemical properties of histones and DNA. A large part of these changes consists in post-translational modifications (PTM) of histones, occurring principally on their amino-terminal tail protruding from the core nucleosome [22, 23]. To date, different PTMs have been identified, among which histone acetylation, methylation, phosphorylation, and ubiquitination are the most studied. The same histone can be modified on different residues simultaneously and a chemical group can be added up to three times on the same residue, leading to a high number of combinations. Many of these modifications are reversible, giving a great plasticity to chromatin and allowing cells to react and adapt efficiently to their environment. These changes alter the interaction of the nuclear proteins with the DNA and release or condense the chromatin to regulate gene expression and to allow various processes including DNA repair, DNA replication, mitosis, and meiosis. All together these PTM constitute the so-called “histone code” [24–27]. Sperm chromatin remodeling is associated with PTM both before and during the replacement of histones by testicular proteins [8, 17–19]. These histone PTM promote protein-protein interactions such as with the double bromodomain-containing protein Brdt that binds acetylated histone H4 resulting in a more relaxed chromatin structure facilitating histone exchange/removal [19, 20]. During sperm chromatin reorganization, histone exchange, which only supports a supercoiled DNA structure, is accompanied by transient DNA strand breaks that function to relax DNA and eliminate free DNA supercoils formed along the process [24, 25, 28, 29]. These DNA strand breaks have been attributed to the activity of topoisomerase II beta (TOPO2ß) that has the ability to remove DNA supercoiling [30]. DNA strand breaks are recognized by poly(ADP-ribose) [PAR] polymerases, PARP1 and PARP2, which coordinate TOPO2ß-dependent DNA decondensation facilitating histone to protamine exchange [31, 32].

Another level of heterogeneity is signified by the observation that in some cell-types nucleosomes contain histone variants [33]. Each canonical histone corresponds to different histones variants, which are homologous proteins of the same gene family. Sequence identity between a variant and its corresponding canonical histones can vary. For example, H3 shares 96% identity with the H3.3 variant and 46% identity with the centromere-specific protein A (CENP-A), another H3 variant. The different primary amino acid sequences confer to histone variants specific structures and their own physicochemical properties. Consequently, histone variants possess different biological functions when compared with canonical histones. It is interesting to note that most histone variants are testis-specific and only expressed in male germ cells during spermatogenesis. This observation highlights rather well the atypical nature of the sperm chromatin organization.

### From a somatic-like chromatin organization to a sperm-specific chromatin organization

During spermatogenesis, germ cells undergo a long process of differentiation to form spermatozoa, highly differentiated cells that consist of a head containing the nucleus and a flagellum allowing them to move towards the oocyte in the female genital tract. This cyto-differentiation process prepares the paternal DNA to be transmitted as a single copy, packaging it tightly to safely withstand the arduous journey in the male and female genital tracts. The transition from a somatic like nucleus to a specific spermatozoa nucleus is a lengthy process that starts during mid-spermatogenesis with the meiosis becoming highly visible afterwards with the great cytological changes accompanying spermiogenesis. Although at the end of the spermiogenesis spermatozoa look completely cyto-differentiated, in reality this does not appear to be the case. In fact sperm structures including the nucleus continue to evolve after spermiation especially during the epididymal transit. The passage from a spermatogonia, a diploid cell, to four haploid spermatids is based on the meiotic process. Like mitosis, meiosis induces the DNA to condense in order to separate homologous chromosomes and chromatids in identical cells. This remodeling of the chromatin during meiosis is allowed by PTM of histones and by insertion of ubiquitous or testis-specific histones variants. These chromatin modifications take part in multiple steps during meiosis including the condensation of chromatids, the repair of the numerous DNA single strand breaks created for the pairing of homologous chromosomes, the sex (or XY) body formation, the substantial activation of transcription during the pachytene stage, and the formation of the kinetochore facilitated by the Cenp-A variant. The precise function of these chromatin modifications during meiosis is still under study (for reviews on these particular aspects see: [34–36]).

The most striking changes of the male germinal chromatin occur during spermiogenesis. In addition to the great changes of their cell morphology, spermatids also undergo major modifications of their nucleus. Accompanying the marked reduction in cell size, the sperm nucleus volume is also profoundly reduced to approximately 1/7th the size of any somatic cell nucleus. This reduction of the sperm head volume serves two distinct purposes; the acquisition of a more hydrodynamic head shape that will determine the cell optimal velocity, and the protection of the paternal DNA from insult by toxic metabolites. In mammals, to achieve this goal, the chromatin is highly condensed from the periphery to the center and from the apex to the base of the nucleus. Chromatin condensation is due to a deep reorganization of DNA-associated proteins. Initially, various histone modifications and the incorporation of histone variants (in particular, linker histone variants: H1t, H1t2, and Hils) is required to open up the chromatin enabling the exchange of histones with transition proteins (Tnp). This is then followed by Tnp replacement with other basic proteins, the protamines (Prm). Among the histone PTM recorded during spermiogenesis, hyperacetylation and ubiquitination occur simultaneously and appear to play an important role in the histone-protamines exchange. H2A and H2B ubiquitination add a large chemical group to the core histone inducing steric hindrance aiding the chromatin opening. In the meantime, the leftover histone de-acetylases (Hdac) from meiosis prophase I, are degraded [37] resulting in the hyperacetylation of H4 and to a lesser extent of H3 in the entire nucleus. In human, the hyperacetylation consists of a phosphorylation sequence of multiple histone residues in a defined manner that precedes and persists during histone-to-protamine exchange. This process of histone hyperacetylation occurs only in species that utilize histone replacement (trout, mollusks, *Drosophila*, rooster, rodents, human), and not in species that conserve histones in their mature sperm cells. Two modes of action for histone hyperacetylation have been proposed which are not mutually exclusive. Firstly, DNA-histone interaction is decreased by histone hyperacetylation, allowing the opening of the chromatin and recruitment of factors and proteins. Secondly, bromodomain proteins can recognize and bind hyperacetylated histones. Notably, the bromodomain testis-specific protein (Brdt), is only expressed in male germ cells during the pachytene, the diplotene, the round spermatid, and the elongating phases [38, 39] which coincide with histone hyperacetylation during spermatogenesis. The binding of Brdt to hyperacetylated H4 induces chromatin condensation, independently of ATP [39, 40]. However, this binding also allows the recruitment of Smarce1 [39], an ATP-dependent SWI/SNF chromatin remodeling complex, which suggests two alternative mechanisms of action for Brdt: an ATP-dependent and an ATP-independent one.

#### The transition proteins

In mammals, hyperacetylated histones are first replaced by transition proteins. This is not the case in all species as for example in mollusks; the histone-protamine exchange does not require an intermediary [41]. Transition proteins (Tnp) are small proteins (between 50 and 140 residues), more basic than histones (but still less basic than protamines) and rich in arginine and lysine. Four Tnp are known in mammals but only Tnp1 and Tnp2 have been well studied. Tnp1 and Tnp2 are encoded by two different single-copy genes composed of 2 exons and an intron. In rodents and humans, the *tnp2* gene is part of a cluster along with *prm1*, *prm2*, and *prm3* genes. This cluster is surrounded by 2 matrix attachment regions (MAR) and involved in the transcriptional regulation of these genes during spermiogenesis [42]. The transcription of these clustered genes and *tnp1*, located on another chromosome, occurs at the same time in round spermatids. The corresponding mRNAs are stored as ribonucleoproteins for 3 to 7 days until translation. The proteins involved in this storage recognize the 3′UTR regions of the mRNAs. Moreover, these transcripts possess a long polyA tail (about 150 nucleotides) partially cleaved (around 50 nucleotides remaining) before translation. TheTnp mRNAs are then translated subsequently the Tnp proteins are phosphorylated at their C-terminus. This phosphorylation is a prerequisite for binding to the DNA. It is subsequently removed to increase the Tnp-DNA affinity and the chromatin condensation [43]. Tnp1 protein is 54 amino acids long, composed of 20% lysine, 20% arginine, and no cysteine (except in boars, bulls, and rams) in a highly conserved sequence between species. Tnp1 is strongly expressed and evenly distributed in the nucleus of spermatids. In vitro, Tnp1 decreases the melting temperature of DNA [44], destabilizes the nucleosome-DNA interaction and relaxes the chromatin on addition to nucleosome-binding DNA [45]. Tnp1 also increases the topoisomerase I activity [46] and stimulates single-strand break repair [47]. In vivo, *tnp1* knock-out in mice did not induce a marked phenotype in sperm nucleus, but was observed to influence fertility [48]. In fact, only 40% of male mice were fertile the litter size was reduced from 7.7 to 1.6 pups/litter when males were mated with females of the same svj129 background. According to the authors, the infertility factor was due to a substantial decrease in sperm motility. In the spermatid nucleus, an abnormal chromatin structure was observed during condensation with the presence of rod-shaped chromatin condensation units in the fine fibrillar chromatin. *In fine*, the chromatin of epididymal spermatozoa was less condensed than in wild-type (WT) mice. The analysis of protein composition in the spermatid nucleus revealed a normal histone withdrawal but an increased incorporation of Tnp2 and a premature production of the Prm2 precursor protein. Moreover, the processing of the Prm2 precursor by cleavage was delayed and stable intermediary forms of Prm2 were detected in cauda epididymal spermatozoa.

Tnp2 is relatively different from Tnp1 in many aspects. This protein is twice as large as Tnp1, with a 117 to 138 amino acids poorly conserved between species. It is composed of 10% lysine, 10% arginine, 5% cysteine, as well as serine and proline. Tnp2 possesses 2 zinc-finger domains in the N-terminal domain and a highly basic C-terminal domain. Its expression levels vary depending on species. In vitro, Tnp2 increases the melting temperature of the DNA and condenses the nucleosome-binding DNA by oligomerization of close DNA strands [49, 50]. In vivo, *Tnp2*-null mice were fertile, however a decrease in litter size was observed (from 7.4 to 3.9 pups/litter; [51]. Epididymal spermatozoa presented flagellar defects and an abnormal chromatin structure that was less condensed than in WT mice, resembling that observed in *tnp1*-null mice. Tnp2 loss was compensated by an increase in Tnp1 expression and maturation defect of the Prm2 precursor (as recorded in *tnp1*-null mice) was observed. The *tnp1*/*tnp2*-null double mutant mice were found to be infertile [52]. These mice showed a great decrease in epididymal sperm counts, motility, viability, and normal morphology. In addition, the chromatin of the few epididymal sperm cells was weakly condensed. Moreover, in vitro fertilization with these spermatozoa revealed poor fertilizing abilities. Thus, this study underlined that Tnp1 and Tnp2 possess some redundant functions, but cannot fully compensate for one another, suggesting some specificity of function. The opposing in vitro properties of Tnp1 and Tnp2 also support these conclusions.

#### The protamines

Transition proteins are replaced in their turns by protamines (Prms). These highly basic small proteins are produced by genes evolutionary derived from an ancestral gene that was also at the origin of the histone H1 gene [53]. Over time, the sequence and the structure of the Prms have much diversified between species [54]. Two Prms, Prm1 and Prm2, have been characterized in mammals. Whereas Prm1 is expressed in all mammals, Prm2 is only expressed in some species such as primates, certain rodents, rabbits, hares, and horses. Although pigs and bulls possess a *prm2* gene, it is not functional. P*rm1* and *prm2* are composed of 2 exons and an intron, as is the case for the *tnp* genes. As indicated already above, in rats, mice, and humans, *prm1* and *prm2* form a cluster with *tnp2* and a third *prm* gene, *prm3. Prm* genes are expressed at the same time in round spermatids and the corresponding mRNAs are stored and inactivated for around 10 days, until the elongating stage, by the same processes than those described for Tnp mRNAs (for reviews see: [43, 55, 56]). It should be noted that *prm3* encodes a small cytoplasmic acidic protein, not involved in the process of spermatid chromatin condensation [57]. As Tnps, Prms are phosphorylated immediately after mRNA translation, during the translocation of the proteins into the nucleus. This PTM is necessary for DNA binding, but is removed afterward, increasing the Prm-DNA affinity and the chromatin condensation. Prm1 is translated as a mature protein of about 50 amino acids, composed of an arginine-rich central domain and of cysteine-rich short domains. The N-terminal tail possesses some serine residues, which are phosphorylation sites involved in Prm1 incorporation in the spermatid chromatin. For its part, Prm2 is synthetized as a precursor protein of about a hundred amino acids. Poly-arginine domains are interspersed throughout the mature Prm2 and the content in histidine is higher than in Prm1 (up to 20 and 5% or less, respectively; [58]. As with Prm1, Prm2 is rich in cysteines and is also phosphorylated immediately after its synthesis enabling it to bind to DNA. The DNA-bound Prm2 is progressively matured by successive proteolytic cleavages of its N-terminus, a process that takes several days, increasing chromatin condensation step-by-step. This maturation process eliminates about 40% of the N-terminal domain of Prm2. In mice and humans, six cleavages are necessary to produce a mature protein of about 60 residues long. However, some of the intermediate products can persist in the mature sperm nuclei [59, 60]. Finally, another important difference between Prm1 and Prm2 is the ability of Prm2 to bind zinc. It is hypothesized that the zinc complex will participate in the final condensation of the sperm nucleus, protecting a number of protamine thiol groups from oxidation and thus limiting the formation of intra- and inter protamine disulfide bonds [61]. However, zinc is particularly enriched in the seminal plasma while disulfide-bridging of protamine thiols is a process taking place during epididymal maturation suggesting that the later is likely to be the prominent process driving the final state of compaction of mature spermatozoa (see below).

#### The final structure of the sperm chromatin

The histone-protamine exchange during the elongating phase of spermiogenesis drastically modifies the structure and the spatial organization of the sperm chromatin. At the end of this process, the sperm chromatin is 6 to 7 times more condensed than in the nucleus of any somatic cells. The high level of sperm DNA condensation almost resembles a crystalline structure, with little room to accommodate even water molecules. The compact structure shields it from mechanical shearing and chemical stressors that may have mutagenic effects. This is particularly important because mature spermatozoa are devoid of any functional DNA repair machinery (see below). Thus, it will be the task of the oocyte after fertilization and prior to the first division of segmentation to repair the paternal DNA in the newly decondensed male pronucleus [62]. Although the processes of sperm nuclear condensation and chromatin reorganization are considered essential, our knowledge of the structural intricacies of the sperm nucleus is scarce. Although it should be pointed out that the sperm nucleus is a highly ordered structure that is well conserved from one sperm cell to another as well as from one individual to another, the organization of the mature sperm nucleus however appears to be species–specific, limiting the ability to translate findings from mouse or bovine to human.

##### The basal unit of the sperm chromatin and its conformation

**Fig. 1:**
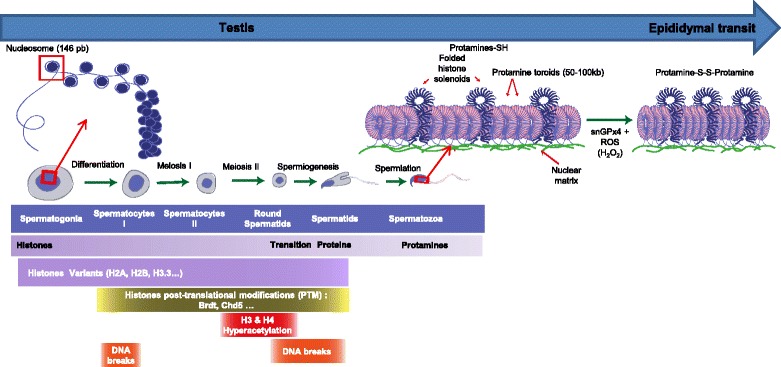
Schematic representation of the testicular and epididymal events leading to the drastic change in sperm chromatin organization. In testes, spermatogenesis permits to transform diploid spermatogonia into haploid spermatozoa. Spermatogenesis can be subdivided into three major steps: a mitotic amplification which ensures the proliferation and maintenance of spermatogonia, a meiotic step in which spermatogonia undergo to form spermatocytes which differentiate into spermatids and a post-meiotic step also known as spermiogenesis which makes spermatozoa. During spermiogenesis, the round spermatids undergo several morphological and biochemical modifications characterized by the acquisition of final nuclear shape and the replacement of somatic type histones by protamines. Histones that organize the DNA (146 bp) into nucleosomes are gradually replaced by testis-specific histone variants, and sudden post-translational modifications (for example hyperacetylation), followed by the replacement of most histones by at first by DNA interacting non histones, then by transitions proteins Tnp1 and Tnp2 and finally by protamines (Prms). Sperm DNA-protamine interaction leads in a unique appearance that involves the coiling of sperm DNA into toroidal subunits, also known as “doughnut loops”, that contain around 50 kb to 100 kb of DNA. At the end of spermatogenesis a fraction of the sperm chromatin is still in nucleosomal arrangement. Remaining histone-containing nucleosomes (folded histone solenoids) punctate the toroidal chromatin structure. In addition, the small linker DNA strands going from one toroid to another are also associated with histones. At some locations, these histone-associated strings of DNA are bound to the sperm nuclear matrix. During post-testicular epididymal maturation of spermatozoa, the nucleus is further condensed by means of intense disulfide bridging. A nuclear enzyme (sperm nucleus glutathione GPx4 = snGPx4) working as a disulfide isomerase uses luminal reactive oxygen species (ROS), essentially hydrogen peroxide (H_2_O_2_) to create inter- and intra-protamine disulfide bounds on thiol groups carried by the cysteine-rich protamines. It further condenses the sperm nucleus and locks it up a condensed state

The nucleoprotamine structure constitutes the basal unit of the sperm chromatin. The Prms and their interaction with the DNA were first studied in salmon and bull [16, 63]. Unfortunately, no detailed crystallographic data are available as the DNA-Prm complex is insoluble. As shown by Raman Spectroscopy, when Prm1 is free in solution, the protein is unfolded, [64]. Prm1 acquires a stable conformation only when it is bound to DNA. Prm1 wraps around the double stranded DNA in one groove of the double helix *via* electrostatic and hydrogen bond interactions with the DNA backbone. Although, most studies conclude that Prm1 binds to the major groove of the DNA [65, 66], some studies report that it also binds to the minor groove [67]. The interaction of one Prm1 *per* turn of the DNA helix covers about 11 bp [68] allowing the DNA to curve in a conformation unique to the sperm chromatin. After binding, due to the presence of numerous cysteine residues contained in protamines, intramolecular disulfide bridges are initially formed to stabilize the Prm1-DNA interaction. Subsequently the intermolecular disulfide bridges formed between Prms trigger the adjacent DNA fibers to come closer for a tighter compaction of the sperm chromatin. Atomic force microcopy studies revealed that the addition of bull Prm1 to a free linearized plasmid DNA on a mica surface prompts its condensation into a toroidal structure, called a toroid [69]. Other experiments showed that around 50 kb of DNA can be coiled into a salmon protamine toroid [70]. Toroids were also observed in native human sperm chromatin [71]. The toroid is therefore considered as the first level of organization of the sperm chromatin (see Fig. 1). Similar properties were found for Prm2-DNA interaction with the addition that zinc ions participate in the interaction. Prm2 binds zinc *via* its cysteine residues with one zinc ion mobilizing 4 cysteine residues. The amount of zinc associated to Prm2 is important since it was reported that there is an equimolar ratio of zinc and Prm2 in human sperm [68]. The zinc-mediated thiol bridges then stabilizes the Prm2 and the sperm chromatin complex [61]. These observations are in agreement with the role associated with zinc in male fertility [61]. It is therefore evident that a fine balance is present between the number of protamine cysteine residues involved in disulfide bridges versus the number of cysteine residues chelated by zinc. This balance is directly linked to the sperm nuclear content in protamines since Prm1 cysteine residues are involved in disulfide bridges while Prm2 cysteine residues are involved in zinc chelation, thus preventing these residues to form disulfides. These observations explain why the sperm nucleus condensation is both sensitive to the redox status and to zinc availability. High levels of zinc will reduce disulfide bond formation leading to a less nuclear compaction while lower concentration of zinc will promote disulfide bridging resulting in a tighter nuclear compaction. Although a tighter sperm nuclear condensation could be perceived as beneficial, it may not be the case since after fertilization a highly condensed paternal DNA will require more time and more energy to be decondensed. Similarly, the redox environment may influence protamine disulfide bridging events consequently sperm nuclear condensation. Thus, we postulate the existence of a fine equilibrium that is critical for optimal sperm nuclear condensation and zinc concentration. The high level of zinc found in the seminal plasma may therefore limit further disulfide bridging events in the sperm nucleus during its journey up to the fertilization site in the female genital tract.

Practically, in high salt conditions and with the use of a reducing agent such as dithiotreitol (DTT) protamines can be extracted from the nucleus of epididymal spermatozoa because of the reduction of the disulfide bridges. This treatment promotes the formation of a halo of DNA loops exiting the sperm nucleus. This halo can be viewed after ethidium bromide staining and its measurement in hamster revealed that average loop length were around 46 kb [72]. This estimate is close to the 50 kb or so of DNA found associated with a toroidal structure of protamines as observed in vitro when using plasmid DNA and salmon protamine [70]. Of note is the fact that with human sperm cells the halo revealed a DNA loop size of about 27 kb [73].

##### The sperm nuclear matrix

The formation of a DNA halo around the sperm nucleus after protamine extraction suggests that the DNA loops arranged around the toroids are attached to an internal nuclear scaffold. Ward and colleagues have proposed that the toroids are associated with a protein-rich nuclear matrix. They showed that the DNA strands linking 2 neighboring toroids are sensitive to nucleases (DNAse I, topoisomerase II β, restriction enzymes…), as is the case for the matrix attachment regions (MAR) in the nucleus of somatic cells [74]. In addition, in the sperm nucleus of hamsters, mice, and humans, the same authors isolated, as part of the nuclear matrix, a protein structure bound to specific DNA sequences [73, 75, 76]. This structure is called the nuclear annulus because of its curved ring shape. It is located at the base of the sperm nucleus at the implantation fossa, the junction between flagellum and sperm head. Further studies revealed that sperm nuclear MARs differ from somatic nuclear MARs. As an illustration, in hamsters, MARs found in the region of the rRNA 5S gene cluster were compared between somatic and sperm cells using fluorescent *in situ* hybridization (FISH) in histone- and Prm-depleted halo nuclei [77]. This gene cluster is organized into a single large DNA loop in liver and brain somatic cells, whereas it forms three short DNA loops in sperm cells. Sperm MARs may therefore not be equivalent to somatic cell MARs. As mentioned above for the *Prm*-*Tnp* gene cluster, the sperm MARs are involved in the regulation of gene transcription in spermatids during spermiogenesis. This may also be the case in the paternal pronucleus of the zygote after fertilization. Additional research in mice underlines the importance of the physical association between these DNA sequences and the sperm nuclear matrix for the paternal pronucleus formation and the first cycle of DNA replication in the zygote [78, 79]. Data suggest that the zygotic origins of replication are located in sperm MARs, as already shown in somatic cells [80, 81].

##### Persisting nucleosomes in sperm

Several reports have shown that histones could be found in mature sperm in variable quantities depending on the species. For example, it was estimated that around 1% of the sperm DNA is still associated with histones in mice, hamster, stallion, and bull spermatozoa [82–84] while it may go up to 10–15% in human sperm [85]. More recent immunoprecipitation studies (yet to be published) narrowed this difference down and reported that in human sperm persisting histones constitute about 5 to 7% of the DNA sequences. This is still substantially more than the other mammals studied to date. The reason for the higher percentage of histones in humans sperm is not yet understood. Some authors suggest an inefficient spermatogenetic program in human as a possible reason. Others postulate that human sperm needs to maintain more paternal chromosomal regions to be readily accessible for the onset of the developmental programme post-fertilization. Initially considered as remnants of an incomplete histone replacement process during spermatogenesis these persisting histones are now considered critical for the early transcriptional reactivation of the paternal genome [86, 87]. This notion is backed by the observation that the persisting histones in sperm can be found in the zygote [88].

The presence of paternal persisting histones after protamine-histone exchange, fertilization followed by decondensation [89, 90] may reveal an important functional role for these proteins in the early embryo development [91, 92].

In mice and human spermatozoa, immunocytochemical approaches reveal localized histones at the periphery of the nucleus as well as in the post-acrosomal and basal domains of the sperm head [93–95]. The basal localization of the histone signal resembles that of the nuclear annulus [75]. This structure is seen as a component of the sperm nuclear matrix, acting as an anchor for the sperm DNA *via* the sperm MARs (localized among toroids) and the histone-rich telomeres [94, 96]. In mouse sperm we recently reported that nuclear domains rich in matrix proteins are also histone-rich regions of lower compaction [95, 97].

The fact that specific locations in the mature sperm nucleus house these histones supports the notion of an ordered process for the maintenance. This is backed by genome-wide analyses including chromatin immunoprecipitation (ChIP) studies, DNA microarrays and high-throughput sequencing revealing organized specific regions in mouse and human sperm. Nucleosomes were found enriched in 2 types of genomic regions. One region concerns large areas of DNA up to 100 kb in length that punctuate the protamine-associated chromatin (see Fig. 1). Ward [98] suggested that these segments of DNA are organized in a less condensed state with a solenoid-like conformation resembling the somatic chromatin. Additionally, high histone levels were found in interspersed short DNA sequences (around 1 kb-long) that correspond to the small DNA linkers bridging toroids together [95, 97, 98]. Interestingly, the latter regions were also reported to be associated with the sperm nuclear matrix [95, 97, 98]. ChIP studies utilizing histone antibodies to recover the histone-associated DNA sequences in mature sperm revealed histones to be significantly enriched at the promoters of: genes coding for microRNAs, genes involved in early embryonic development (e.g. transcription factors, HOX genes, signaling protein, etc.…), genes subjected to genomic imprinting, and genes involved in spermatogenesis [99]. However, conflicting data from different groups showed that persisting histones were associated with intergenic sequences outside of the gene regulatory regions [95, 100]. To date, there is no consensus as to what specific DNA sequences in sperm are associated with nucleosomes. This is essentially due to the way DNA sequences were recovered from mature sperm during the ChIP assays. Due to the extreme difficulty in recovering mature sperm DNA associated with the intrinsic properties of the compacted nucleus, strong reducing conditions are required to obtain the halo sperm phenotype followed by microccocal nuclease (Mnase) digestion to retrieve the long loops of DNA exiting the sperm nucleus. The soluble DNA fraction is then separated from the insoluble portion that also contains the nuclear debris. This operation leads to the recovery of sperm DNA sequences associated with histones which mainly corresponds to the solenoid regions embedded within the toroids of protamines (see above). Following this type of sperm DNA retrieval protocol it is not possible to recover the sperm DNA associated with the nuclear matrix as it is discarded during the preparation. However, with more aggressive sperm DNA retrieval conditions such as sonication, it is possible to obtain some of the histone-associated DNA sequences attached to the sperm nuclear matrix. The use of different extraction techniques may thus explain the present discrepancy in the literature of sperm DNA histone-associated sequences in the species that have been investigated.

Another important question is why peripheral regions of the mature sperm nucleus maintain association with histones? Due to their lower state of compaction and their peripheral localization, these nuclear domains will be the first ones to be susceptible to DNA damage from external stressors. So what is the evolutionary reason for the maintainance of such regions of fragility? One reason could be that these peripheral regions are the first ones targeted by the oocyte-driven centripetal decondensing processes of the sperm nucleus after fertilization. Accordingly, these regions may have to be retained in a pre-decondensed state either to facilitate the decondensation process or/and to remain transcriptionally active very early in the zygote developmental program. Regardless of the reasons, the paternal DNA present in these peripheral less tightly packed regions of the sperm nucleus will be more likely to incur damage. This observation has been verified experimentally in transgenic mouse models where it was observed that the regions sensitive to high post-testicular oxidative damage match the distribution of nucleosomes [95, 98].

A more detailed analysis of histone variants in these loci, where histones are maintained, showed a precise distribution of these epigenetic marks. For example, the testis-specific H2B variant (TH2B) was found enriched in the promoters of genes involved in sperm cell maturation, function, capacitation and fertilization, but never in the promoters of genes controlling embryonic development [89]. As another example, the H2A.Z variant was mainly found in pericentromeric heterochromatin domains [101]. Conversely, the promoters of developmental transcription factors genes were found enriched in H3K4me2 marks (transcriptionally permissive marks consisting in dimethylation of histone H3 on lysine 4) while H3K9me3 marks (transcriptionally repressive marks consisting in trimethylation of histone H3 on lysine 9) were not found localized near genes, but in pericentromeric genomic regions [99]. In conclusion, histone content and PTM of these paternal-borne nucleosomes represent distinct epigenetic characteristics that are unique to the sperm nucleus. Notably, protamines (Prm1 and Prm2) also carry multiple PTM that have led some authors to propose that like the somatic cell “histone code”, there might also be a sperm “protamine code”, unique to the sperm nucleus [102]. Thus, PTM of sperm persisting histones as well as protamines may constitute a complex epigenetic signature driving embryo development and potentially transgenerational inheritance, as persisting paternal histones may be transmitted to the next generation.

##### The sperm chromosomal organization

Additional studies provide further evidence for the highly conserved sperm chromatin organization at the chromosomal level in the sperm cells of the same individual but also across the individuals of the same species. Studies over the last two decades with the FISH technique applied to sperm cells revealed distinct positioning of the chromosomes in the nucleus. Indeed, in humans, the centromeres of chromosomes are localized in the center of the sperm nucleus, whereas the telomeres are at the periphery [103] where they can dimerize. The use of fluorescence in situ hybridization (FISH) probes for each arms of the same chromosome showed the q- and t-arms co-localize in the same region of the sperm nucleus. It was proposed that the two arms of a chromosome interweave or juxtapose in an anti-parallel manner. In this way, each chromosome has a hairpin structure on a center-periphery axis [104]. Moreover, the chromosomes are not tangled and their position relative to each other appear to be specific occupying a precise location in the sperm nucleus. Statistically, the relative localizations of autosomal and sex chromosomes are preserved between sperm cells of an individual and within the species [105–107]. In humans, the organization of several chromosomes (namely chromosomes 17, 1, X, 19, Y) was partially established along the antero-posterior axis of the sperm nucleus [107, 108], however, to date there is no complete location mapping of all the chromosome in one species.

## Sperm nuclear/DNA alterations have many faces

### Chromatin defects

There is a growing body of evidence supporting the hypothesis that sperm chromatin integrity is an important factor in determining reproductive success. Disruption and alterations of sperm chromatin organization have been associated with various developmental impairments as well as post conception issues such as increased miscarriages, increased perinatal mortality and increased susceptibility to pathologies in the progeny [109–112]. As already described, chromatin compaction in sperm nucleus is a long and complex process leading to a nucleus approximately 7 times smaller than a somatic cell nucleus. Sperm nuclear compaction involves a major and a minor step. The major step involves the replacement of histones by protamines during spermiogenesis and, the “minor” step (relative to the modifications introduced to the sperm nucleus), occurs during epididymal maturation. The latter step involves inter- and intra-protamines cross-linking through formation of disulfide-bonds by numerous thiol groups present in protamines. In each of these compartments (testicular or epididymal) defects may alter sperm nuclear organization and essentially its compaction.

#### Chromatin defects occurring during spermatogenesis

There are numerous ways in which the sperm nuclear structure could be compromised frequently resulting in local or global abnormal sperm nuclear condensation. When local, it is often associated with nuclear vacuoles usually visible at high magnification (above x6000) depending on their size and number. It is now well accepted that these sperm head vacuoles are indeed nuclear in nature and relate to local impairment in nuclear condensation [113, 114]. Local defects in protamination are likely the cause, however, even with the phenotype characterized, the mechanisms involved are not well understood and requires further research. To avoid the selection of spermatozoa with vacuoles for IntraCytoplasmic Sperm Injection (ICSI) procedure, some clinicians now recommend the use of differential interference contrast microscopy (also called Normarski contrast) that allows the observation of live sperm at high magnification. The use of the Motile Sperm Organelle Morphology Examination (MSOME) technique allows the detection of sperm head vacuoles that otherwise would go unnoticed at the regular magnification (x300) used in routine for the selection of sperm for ICSI. However, the use of this protocol in improving ICSI outcome is not yet confirmed due to the paucity of comparative studies [115–121]. Indeed only a few studies comparing the results of IMSI (Intracytoplasmic morphologically selected sperm injection) with regular ICSI have been reported with the blastocyst implantation rate, miscarriages and birth rates as the end points. The sole indication of the presence of chromatin defects that prevails to date is when clinicians face recurrent implantation failures while performing ICSI [122]. The drawback of such sperm selection protocols is the time they require and the exposure of sperm to bright light and media that may lead to oxidative DNA damage.

In addition to these local alterations of the sperm nuclear condensation, there are instances of global sperm nuclear decondensation. These may have several causes such as defective protamination and excessive DNA fragmentation. The latter may be due to unrepaired meiotic strand breaks, mechanical shearing during the cytodifferentiation step at spermiogenesis, or/and the result of oxidative damage essentially in response to environmental conditions (chemical and physical stressors). To better understand the histone to protamine exchange process and the importance of an optimal sperm chromatin organization and compaction on male fertility, different mutant mice were generated. Mice deleted for the two poly(ADP-ribose) polymerases (Parp1 and Parp2), essential proteins involved during the replacement of histone by Prm, are infertile and show increased amount of histones in spermatocytes along with reduced sperm chromatin condensation [123, 124]. Mice knockout for Brdt (Bromodomain-containing protein) which recognizes hyperacetylated histones allowing their replacement by transition proteins (Tpn) are also infertile. They present a severe reduction both in sperm counts and sperm motility associated with high percentage of sperm morphological abnormalities including misshaped heads and failure of nuclear condensation [125]. A similar phenotype was observed in the chromodomain helicase DNA binding protein 5 (Chd5) mutant mice. This was not a surprise since Chd5, similarly to Brdt, orchestrates histone replacement, histone H4 hyperacetylation, histone variant expression and nucleosome eviction [126]. It is worth noting that CHD5 was found highly expressed in the human testis during spermiogenesis and that low CHD5 expression was associated with some human infertile situations [126]. Another knockout model, the *rnf8* ^*−/−*^ mouse, associates sperm chromatin compaction defect with male infertility. Rnf8 is a ubiquitin E3 ligase that ubiquitinates in particular histones H2A and H2B. This PTM appears to promote histone H4K16 acetylation, a critical modification for the replacement of histones by protamines during spermiogenesis. As a consequence, rounded germ cell nuclei of *Rnf8*-deficient male mice contained histones but no Prm leading to severely impaired sperm chromatin compaction. In addition, Tnp1 and Tnp2 levels were dramatically reduced and a mild elevation of germ cell apoptosis was recorded in both testes and epididymis. *rnf8* ^−/−^ sperm also displayed abnormal rounded heads and retained residual cytoplasm [127]. Likewise, Tnp1- and Tnp2-null double mutant mice present with fertility problems associated with abnormal sperm chromatin condensation due to incomplete protamination, a decrease in sperm count as well as a decrease in sperm motility [128]. With respect to the mammalian protamines Prm1 and Prm2, we have seen above that the incorporation of these two proteins into the sperm chromatin is strictly regulated, resulting in a species-specific, tightly controlled Prm1/Prm2 ratio. In human sperm the ratio of PRM1/PRM2 is approximately equal (1:1) in fertile men whereas it was found to be displaced in some infertile situations [129–131]. Patients with altered P1/P2 ratio were shown to be more likely to display decreased sperm concentration, decreased motility, a higher frequency of abnormal sperm morphology and an elevated level of DNA damage. In mice, the knockout of Prm1, Prm2 or both is associated with infertility and non-functional spermatozoa showing abnormal morphology, reduced sperm counts and motility as well as increased DNA fragmentation [57, 132–134]. Recently, Prm1-deficient female chimeric mice carrying Prm1-deficient oocytes were generated. These mice successfully produced Prm1(+/−) male mice. Via in vitro fertilization (IVF) healthy Prm1(+/−) offspring were produced demonstrating that spermatozoa lacking Prm1 can fertilize and produce viable embryos. However a detailed survey of the offspring showed midification in expression profiles [135].

#### Chromatin defects due to chromosomal abnormalities

Sperm aneuploidy originates from segregation errors during the meiotic divisions of spermatogenesis, though the exact causes of sperm aneuploidy are unknown. Perrin et al. [136] found that men with structural chromosomal abnormalities (reciprocal translocations, Robertsonian translocations and pericentric inversions) had higher rates of DNA fragmentation than men without abnormal karyotypes. DNA fragmentation was significantly correlated with the percentage of aneuploidy in chromosomes X, Y, 13, 18 and 21 [137]. Muriel et al. [138] and Enciso et al. [139], have confirmed these findings and shown that sperm aneuploidy may lead to an increase in sperm DNA damage. This is consistent with the fact that sperm DNA integrity partly depends on the structural organization of the sperm nucleus and that chromosome alterations may lead to local architecture modifications [140]. To date, it is not that clear whether DNA damage and aneuploidy in sperm are causally linked or associated by a common mechanism of damage, or if it is just the consequence of a disrupted nuclear architecture [141]. It should be noted that recent findings report that chromosome structural aberrations (disomy, translocation) found in the offspring can be also caused by the oocyte attempt to repair DNA alterations accumulated on sperm DNA after spermatogenesis [142].

#### Post-testicular chromatin defects

Leaving the testes, spermatozoa are not completely mature both at the structural and functional levels. Amongst the numerous modifications that occur on spermatozoa while going down the epididymal tubule up to the storage compartment one concerns the completion of the sperm nucleus compaction. We and others have demonstrated that during the epididymal journey, the sperm nuclear protamines rich in cysteine thiol-containing residues are modified by oxidative events resulting in the formation of intra and inter-protamine disulfide bridges. This nuclear process requires the activity of an enzyme located in the sperm nuclear compartment, the snGpx4 protein (sperm nucleus glutathione peroxidase 4, [143]) that uses hydrogen peroxide (H_2_O_2_) to make disulfide bonds, thus working as a disulfide isomerase. There is now a large body of evidence showing that a finely tuned level of oxidation is required to further condense the sperm nucleus. Absence of *sngpx4* induces a delay in post-testicular sperm nuclear compaction with abnormal chromatin condensation and sperm head fragility when cells are eventually challenged by mild reducing conditions, resulting in a giant head phenotype [143, 144]. We have shown earlier that in mouse an epididymal luminal scavenger, the glutathione peroxidase 5 protein (Gpx5) is critical in this post-testicular disulfide bridging process since it contributes to fixing the optimal H_2_O_2_ concentration in the epididymal fluid [145]. Consequently it also determines the optimal level of disulfide bridging on the sperm nucleus [146]. When Gpx5 is absent (in *gpx5* ^*−/−*^ animals) it results in DNA oxidative damage, mainly to the sperm nucleus, a cell compartment that is difficult to protect even though the epididymis of *gpx5*-deficient animals does it best to limit the increase of luminal oxidative attacks on the sperm plasma membrane [145]. Sperm DNA oxidation was associated with reproductive failures including an increase in miscarriages, an increase in abnormal embryonic development and an increase in perinatal mortality. When both *sngpx4* and *gpx5* were knocked out we recorded cumulative effects on sperm cells that show both nuclear susceptibility to decondensation and nuclear oxidation [144]. Therefore, when the testis is not at the origin of the defect, the sperm nuclear condensation can also be challenged in its post-testicular life especially during epididymal transit where oxidative processes are at work to complete it [146, 147]. Any disruption of the oxidative balance in or around sperm cells in their post-testicular life may thus affect the level of nuclear condensation. Oxidative stress, but also reductive stress, may end-up having the same effects on the sperm nucleus state of condensation. Excessive oxidation while it may serve nuclear condensation at first will lead to DNA fragmentation, resulting ultimately in decondensation [148]. Reductive stress will destroy the disulfide bridges sticking the nuclear protamines together and therefore will result in decondensation of the sperm nucleus [148].

### DNA damage

The most well known and measured sperm DNA damage is DNA fragmentation or DNA strand breaks (DSB). DSBs, either single strand break (SSB) and double strand breaks (DSB), can occur during sperm generation or/and during the post-testicular journey. In the testicular compartment it may be the result of apoptosis, defective DNA repair mechanisms, mechanical shearing as well as the result of oxidative attacks. In the post-testicular compartment DNA strand breaks essentially originate from oxidative attacks.

#### Germ cell apoptosis and DNA fragmentation during spermatogenesis

Apoptosis is a major feature of male germ cell development. Apoptosis is used as a mechanism for the removal of damaged germ cells from seminiferous tubules so that they do not continue to differentiate into spermatozoa. During spermiogenesis, Sertoli cells are responsible for the induction of apoptosis in 50 to 60% of all germ cells that enter meiosis. Germ cells marked with apoptotic markers of the Fas type are eliminated by the Sertoli cell *via* a phagocytic process [149, 150]. However, this mechanism may not always operate efficiently and a variable percentage of apoptotic germ cells enter the process of sperm remodeling and appear later in the ejaculate. They present DNA strands-breaks associated to the apoptotic process (=abortive apoptosis). Apoptosis during spermatogenesis has been suggested to play a role in the etiology of spontaneous male infertility in light of the excessively high numbers of apoptotic germ cells observed in the testes of some infertile males [151]. In addition, apoptotic markers including caspase activation and phosphatidylserine exteriorization have been detected in mature spermatozoa from infertile males [152]. Besides the apoptotic process there is another way by which DNA fragmentation may occur during spermatogenesis. During spermiogenesis it was shown that chromatin packaging requires endogenous nuclease and ligase activities to create and ligate nicks that facilitate the protamination step. McPherson and Longo [153] hypothesized that the presence of high level of DNA nicks in ejaculated sperm may be indicative of impaired spermiogenesis [153]. These nicks are thought to provide relief of torsional stress to support chromatin arrangement during the displacement of histones by the protamines [154]. If not repaired completely in a timely manner they may affect sperm chromatin packaging and render spermatozoa more susceptible to post-testicular damage. As an illustration, mice deficient for poly(ADP-ribose) show a high level of unrepaired DNA nicks that are associated with male infertility [31].

#### Sperm DNA fragmentation induced by Reactive Oxygen Species (ROS)

ROS are natural products of cellular metabolism. In physiological concentrations, sperm cells require ROS at different moments of their life. During epidiymal maturation ROS (especially H_2_O_2_) participates in the processes of sperm maturation (disulfide bridges on sperm proteins). ROS are also required for successful oocyte fertilization acting as second messengers in the capacitation processes including hyperactivated motility and acrosomal exocytosis. However, when ROS generation exceeds ROS recycling it contributes to a large proportion of instances of male infertily [155, 156]. ROS target the polyunsaturated fatty acid (PUFA) rich sperm plasma membrane altering membrane fluidity and mitochondria functions resulting in impaired mobility and decreased fusogenic capacity with the oocyte. ROS, especially hydrogen peroxide (H_2_O_2_) may also reach the sperm nucleus leading to oxidative DNA damage that may lead to mutagenic effects which may be transmitted. There are many common situations that may lead to sperm exposure to ROS whether it is secondary to aging, environmental factors (exposures to toxicants, drugs, UV, ionizing radiations, heat…), pathological situations (infection, inflammation, metabolic disorders….), lifestyle (unbalanced diet, smoking, alcohol addiction…) [157–162]. In addition, sperm exposure to ROS may happen during assisted reproductive technologies (ART) procedures for example during sperm cryopreservation, exposure to culture media or sperm handling during selection processes, especially for ICSI (intracytoplasmic Sperm Injection) [163]. As we have seen above, because ROS and especially H_2_O_2_ are such important contributors to the completion of the sperm nucleus compaction during epididymal transit and beyond, it is no surprise to find them involved in nearly all situations of defective spermatozoa.

Oxidative alterations of cellular components are a very common problem of aerobic cells which are usually well–equipped in enzymatic and non-enzymatic primary and secondary antioxidants to deal with it. Oxidative alterations can affect the nuclear compartment of any cells, especially because reactive oxygen species such as H_2_O_2_ can freely pass through plasma membranes. When this happens and when ROS reaches the nucleus it may be at the origin of modified bases, abasic sites, chromatin protein cross-linking and DNA strand breaks (both single and double) depending on the intensity of the oxidative attack [148]. In any somatic cell as well as in an oocyte, the base excision repair (BER) pathway will replace these oxidized bases by non-oxidized bases correcting the alterations. Mature sperm cells cannot do that as they have been shown to lack the necessary equipment [62]. Only the first player of the Base Excision Repair (BER) pathway, the 8-oxoguanine DNA glycosylase 1 (Ogg1/OGG1) was shown to work in rodent and human spermatozoa [62]. OGG1 activity marks the oxidized base to be removed [62]. Sperm cells will then rely on the oocyte BER equipment that will correct the paternal genome after fertilization upon the decondensation step of the male pronucleus and before the first division of segmentation. The consequence of sperm DNA damage with respect to normal embryo development is therefore the result of an equilibrium between the extent of nuclear oxidation and the DNA repair capacity of the oocyte [164, 165]. It has been shown that the zygote responds to sperm DNA damage through a non-apoptotic mechanism that acts by slowing paternal DNA replication and ultimately leads to the arrest of embryonic development [164, 166–168]. After induction of DNA oxidative damage on Rhesus sperm using xanthine and xanthine oxidase, Burruel et al. [169] have shown that ICSI-produced embryos present severe fragmentation, multi-nucleation, and early cell arrest essentially around the four-cell stage. Because of this inability to repair its DNA the mature spermatozoa is very sensitive to DNA oxidative alterations. Paradoxically, mature spermatozoa are prone to suffer oxidative attacks because they harbor a peculiar plasma membrane rich in polyunsaturated fatty acids (PUFA) that is highly susceptible to ROS. When oxidized in situations with an excess of ROS or weakness in the antioxidant protective activities, these PUFA will amplify the generation of ROS in a vicious oxidative stress circle [170]. In addition, even-though they are most sensitive to oxidative stress we have seen above that mature spermatozoa are physiologically exposed to ROS. We, and others have demonstrated that part of their post-testicular (ie. epididymal) maturation step utilizes a finely tuned concentration of H_2_O_2_ to complete the condensation of the sperm nucleus *via* disulfide bridging events on the thiol-containing protamines. How relevant sperm DNA oxidation is with respect to male infertility is difficult to say at this stage in the absence of clinical trials in which the level of sperm DNA oxidation is correlated with reproductive success. However, there are recent reports suggesting that it is a major concern since it was shown that over 60% of male entering ART programs present medium to high levels of sperm DNA oxidative alterations [171]. The oxidized base adduct, 8-hydroxy-2′-deoxyguanosine (8-OHdG) has been used in studies to demonstrate that oxidative DNA damage is significantly elevated in the spermatozoa of patients attending infertility clinics [172]. Robust clinical trials are necessary to correlate reproductive success with sperm DNA oxidation.

A common belief is to think that sperm nuclear fragmentation is always associated with sperm nuclear oxidation. Although this is true when the oxidative stress around sperm cells is high, in many situations a mild oxidative stress will not lead to sperm DNA fragmentation. Therefore, one cannot simply say that there is no sperm DNA oxidative damage by assessing the level of sperm DNA fragmentation. This is clearly demonstrated in transgenic animal models having medium sperm DNA oxidative alterations that are not associated with DNA fragmentation [145]. In these models the level of sperm DNA oxidation is sufficient to lead to reproductive failures when transgenic males are crossed with WT females [145]. The reproductive problems recorded ranged from increased miscarriages, increased abnormal development and increased perinatal mortality, all classical issues in reproductive defects both in natural and artificial reproduction.

### Other discrete sperm nuclear alterations

Beside the above-mentioned issues, there are other more subtle sperm DNA/nuclear alterations that may affect reproductive success and the health of the progeny. Apart from oxidation, sperm DNA as in somatic cells may suffer damage affecting nitrogenous bases. While somatic cells are able to repair to a certain extent these altered bases, the highly condensed sperm chromatin cannot. These chemical modifications of nitrogenous bases (alkylation or oxidation) affect mainly the guanine bases. It includes the alkyl DNA adduct known as N7-methyldeoxyguanosine (N7-MedG). N7-MedG is a biomarker signifying environmental exposure to alkylating agents either from diet (industrially processed and preserved foods), well-cooked meat, smoking or various alkylating drugs. It was reported that men diagnosed with male factor infertility had significantly higher mean levels of N7MedG in their sperm DNA [173]. Logistic regression analysis showed that N7-MedG levels were significantly negatively associated with the proportion of oocytes successfully fertilized irrespective of the method of fertilization used IVF or ICSI intra-cytoplasmic sperm injection [173]. Likewise, acrylamide exposition induces formation of N-7(2-carbamoyl-2-hydroxyethyl-) guanine (or N7-GA-Gua). Such situation was not associated with a decrease in fertility but an impact on future generations was reported [174].

The sperm epigenetic information with its multiple carriers: chromatin, DNA and the vast array of coding and non-coding RNAs (small and long) may also be the subject of alterations because of both genetic reasons and in response to environmental situations [175, 176]. Changes in sperm persisting histone acetylation, DNA methylation, and sperm-associated microRNAs were recently shown to be causal for offspring’s diseases in later life, suggesting that a paternal programming does exist [177]. Jenkins et al. [178] found that age-related changes in sperm DNA methylation are located at genes previously associated with schizophrenia, bipolar disorder [178–180] and lesser intelligence. In a recent review [179] the evidence of sperm histone and protamine packing involvement on epigenetic inheritance was reaffirmed, the proper compaction of sperm DNA being necessary to avoid access to nucleases and appropriate transcriptional and translational activities. Any alteration in sperm histone retention would affect DNA integrity and could lead to chromatin rearrangements in developmental loci and genes with impacts on embryo development [166, 167]. In addition, very recent data have shown that sperm microRNAs as well as sperm small non-coding RNAs contents may respond to environmental conditions ([181, 182], Chu C. personal communication). After transmission to the oocyte through fertilization these different epigenetic characteristics may modify the next generations [176, 183].

### Consequences of sperm nuclear damage

**Fig. 2:**
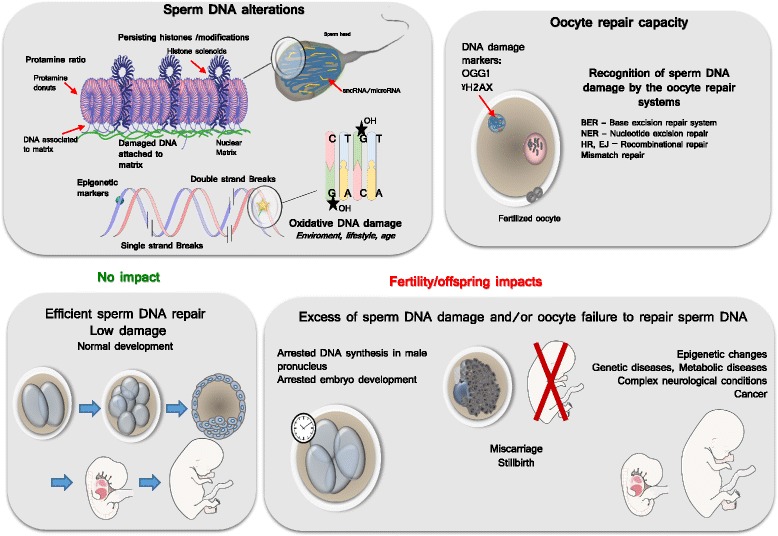
Schematic representation of some aspects of sperm DNA damage and their putative consequences if not repaired. The upper left insert illustrates the major alterations suffered by the sperm DNA from strand breaks, alterations of epigenetic marks and base oxidative damage (such as the 8-OHdG residue. It also show the preferential sites where such alterations preferentially occurs corresponding to the genomic regions of lesser compaction still in nucleosomal organization (histone solenoids within the protamine-containing toroidal donuts, and the small DNA linkers associating protamine donuts. The upper right insert depicts the oocyte repair capacity that has the task to repair the paternal DNA. The lower left insert shows a harmonious development while the lower right panel illustrates some of the classical consequences of oocyte failure/inability to repair the paternal DNA alterations

Sperm nuclear alteration has multiple consequences among which the detrimental impact it has on the success of reproduction may not be the biggest problem. As painful and frustrating it is for a couple having difficulties to conceive because of detrimental sperm nuclear factors, the acceptance of sterility might be somehow easier to deal with than the possibility of having an abnormal embryo development or having a child plagued with a debilitating/incapacitating/life threatening disease (Fig. 2).

The consequence of sperm nuclear/DNA damage with respect to normal embryo development is the result of an equilibrium between the damage contained in the sperm and the DNA repair capacity of the oocyte [165]. As an example oxidative DNA lesions (8-OHdG residues) may lead to transversion mutations (G-C to T-A) which can alter gene expression if not repaired by the oocyte BER enzymes prior to zygote S-phase [184]. The zygote responds to sperm DNA damage through non-apoptotic mechanisms that act by slowing paternal DNA replication. Ultimately, this leads to an arrest in embryonic development [167, 168]. As said above already, the induction of oxidative damage on Rhesus sperm prior to their use in resulted in severe fragmentation, multi-nucleation, and cell arrest before the eight-cell stage, mainly at the four cell stage [169]. This demonstrates well how detrimental to an optimal embryonic program sperm DNA oxidative alterations can be. Kocer et al. [95] found that not every mouse sperm chromosome were susceptible to DNA oxidative damage. The chromosomes or chromosomal regions that are more peripheral in the mouse sperm nucleus were found more susceptible to oxidative damage [95]. In particular, the Y chromosome was found very vulnerable to oxidative attacks. Because of its intrinsic characteristics (rich in repetitive sequences, not repaired by homologous recombination, poorly corrected by the oocyte BER pathway after fertilization) the Y chromosome is at risk of transmitting *de novo* mutations to the progeny that may lead to infertility and an increased risk of cancers in the offspring [185].

Fernandez-Gonzalez et al. [168] evaluated long-term consequences on health and behaviour of mice generated by ICSI using DNA damaged sperm and the results were disturbing. Early effects were a delay in male pronucleus demethylation, lower birth rates while long term effects were lung and dermis tumors, premature aging and death when compared to controls. In addition, they noted that male mice displayed higher emotional reactivity compared with control mice, while female mice showed memory deficits, hypolocomotion, anxiety, and increased adiposity. Similar results on adiposity alterations and glucose metabolism where found [186], by inducing oxidative damage on mice sperm DNA prior to ICSI. One obvious consequence of sperm DNA damage is the increase risk in the frequency of *de novo* mutations in the offspring. In a recent study [187], induced DNA damage (nucleotide modifications, single and double strand breaks) in mice sperm via the exposure to ionizing radiation revealed that the number of *de novo* mutations and clustered mutations were higher in the exposed group when compared to control group. This type of sperm DNA alterations has been associated with autism spectrum syndrome in humans [179, 188]. Similarily, sperm DNA damage was suspected to be involved in behavior or mental alterations in mice [168].

## Evaluation of sperm nuclear integrity

Regardless of controversies, it has been exhaustively shown that paternal gamete integrity has a huge impact on fertility, pregnancy rates and offspring health, independently of the ART used, making the determination of reliable sperm DNA quality test essential as a predictor of health and pregnancy success. Despite the fact that DNA damage in human sperm cells has been linked to a variety of important altered outcomes such as subfertility & infertility [189], an increased incidence of abortions and an increased risk of diseases in the offspring [190] it is still rather difficult to have a clear picture of how detrimental to reproductive success and to the progeny sperm DNA/nuclear damage is. The main reason is that “sperm DNA/nuclear damage” covers a large variety of situations, as we have seen above, is largely underestimated essentially because not routinely monitored. Even though nowadays there is a rather solid consensus among scientists and clinicians that sperm DNA integrity is a determining factor for reproductive success there is a very limited number of infertility clinics monitoring it. There is no shortage of scientific evidence and as an example in the last 20 years we observe more than 1500 reports dealing with sperm DNA fragmentation alone with more than a thousand of these in the last 6 years, indicating how pertinent this topic has become. Still, sperm DNA integrity is rarely part of the routine clinical evaluation of the male partner in the infertile couple. Why is it that so difficult to have international health agencies enforce or at least recommend that it should be the case? The main reason for this inconsistency is that there is no strong consensus as to which of the currently available assays allowing some kind of evaluation of sperm DNA/nuclear integrity is/are predictive of reproductive success. For that reason, international recommendations for pre-ART male check-up still indicate that a classical investigation to evaluate the fertilizing ability of the male partner is based on sperm count, sperm morphology and gross sperm mobility despite that none of these parameters address directly the important criteria of the quality/integrity of the sperm nucleus.

Over the years, a number of tests have been made available to assess sperm nuclear/DNA integrity. Globally, they fall in two categories, direct assays or indirect assays.

### Indirect assays

These indirect assays essentially evaluate the sperm nuclear compaction. They include the:

- Chromomycin A3 (CMA3) assay: CMA3 is a fluorochrome that competes with protamines in the sperm nucleus giving an indirect indication of the protamine level and consequently of the sperm nucleus condensation [191].
- Toluidine blue and aniline blue staining assays: these are common assays used to assess sperm chromatin structure and packaging. With toluidine blue, sperm nuclei with normal chromatin compaction are stained in light blue while those with abnormal chromatin condensation are stained in deep violet (purple, [192]). This is essentially due to the facility with which the intercalating dye penetrates the sperm nucleus. Aniline blue is another dye commonly used to assess sperm nuclear condensation. Anilin blue shows affinity for histones. If sperm cells are stained it indirectly reveals protamination defects and the presence of high level of persisting histones [192, 193].
- MSOME (or Motile Sperm Organelle Morphology Examination) is based on a morphological analysis of isolated motile spermatozoa in real-time at high-magnification (up to x6600). MSOME is able to identify not only conventional morphological sperm alterations with a definition close to that of scanning electron microscopy, but also more specifically sperm head vacuoles, considered as nuclear defects [194–197]. Although MSOME may be helpful in revealing abnormal sperm head morphology and the presence of nuclear defects, it is associated with a high exposure of sperm cells to light during microscopy selection that is known to promote oxidative damage.

### Direct assays

These direct assays essentially evaluate the level of sperm DNA fragmentation and the level of DNA oxidation. They include the:

- Acridine orange (AO) assay. With acridine orange, a normally condensed sperm nucleus shows green fluorescence while sperm cells with abnormal chromatin condensation are red-orange [198]. Acridine orange is an organic compound used as a nucleic acid-selective fluorescent cationic dye. It is cell-permeable and it interacts with DNA and RNA by intercalation or electrostatic attractions, respectively. When bound to double strand DNA, it has an excitation maximum at 502 nm and an emission maximum at 525 nm (green) similar to fluorescein. However, when single strand DNA is bound by A0 there is a wavelength shift since the excitation and emission maxima are reached respectively at 460 nm (blue) and 650 nm (red). When mild acid denaturation of sperm DNA is carried out AO binds to dsDNA (green fluoresecnce, corresponding to non-denatured DNA) and to ssDNA (red fluorescence corresponding to denatured DNA since only DNA with single stranded breaks can be denatured with acid). A correlation was shown between the ratio of red to green fluorescence and sperm nuclear abnormalities such as morphological defects and lack of DNA condensation [198]. A flow cytometry development of AO staining is commercially known as the Sperm Chromatin Structure Assay (SCSA^R^). It is the pioneering assay for the detection of sperm DNA damage and altered proteins in sperm nuclei via flow cytometry of acridine orange (AO) stained sperm. The SCSA® is considered to be the most friendly, precise, repeatable, time- and cost-efficient, precise DNA fragmentation assay with the most data accumulated. It provides very unique, dual parameter data (red *vs* green fluorescence) on a 1.024x1.024 channel scale, not only on DNA fragmentation but also on abnormal sperm characterized by a lack of normal exchange of histones to protamines [199, 200]. It is also the only fragmentation assay with an accepted clinical threshold (DNA fragmentation Index or DFI) for placing a man at risk for infertility.
- TUNEL assay (or Terminal deoxynucleotidyl transferase-mediated deoxyrudine triophosphate Nick End Labeling) uses an independent DNA polymerase called terminal deoxynucleotidyl transferase (TdT) that non preferentially adds deoxyribonucleotides in 3′ hydroxyl (3′-OH) of single or double DNA strand breaks [201]. Although the TUNEL assay is a rather solid assay, in some situations it may lead to an underestimation of the true level of DNA breaks since it will not take into account the abasic sites (promoted by oxidative stress) because of the absence of 3-OH protruding ends.
- Comet assay or single-cell gel electrophoresis. It is a simple and sensitive technique to measure DNA breaks in individual sperm. During this procedure, sperm cells are embedded in a thin layer of agarose on a microscope slide and lysed with detergent under high salt concentration conditions. This process removes protamines and histones allowing the nucleus to form a nucleoid-like structure containing supercoiled loops of DNA. Alkaline pH conditions relaxing double-stranded DNA, and subsequent electrophoresis result in the migration of broken strands towards the anode, forming a comet tail, when observed under fluorescence microscopy. The amount of DNA in the head and tail is reflected by its fluorescent intensity. The relative fluorescence in the tail compared with its head serves as a measure of the level of fragmented DNA [202].
- SCD or sperm chromatin dispersion assay. This assay is based on the principle that sperm with fragmented DNA fail to produce the characteristic halo of dispersed DNA loops that is observed in sperm with non-fragmented DNA, following acid denaturation and removal of nuclear proteins [203].
- DBD-FISH or DNA breakage detection fluorescence *in situ* hybridization [204]. This assay allows *in situ* detection and quantification of DNA breaks in single cells. Cells embedded within an agarose matrix on a slide are exposed to an alkaline unwinding solution that transforms DNA strand breaks into ssDNA (single strand DNA) motifs. After neutralizing and protein removal, ssDNA are accessible to hybridization with whole genome or specific DNA probes. The probes highlight the chromatin area to be analyzed. As DNA breaks increase in the targeted region, more ssDNA are produced by the alkaline solution and more probes hybridize, resulting in an increase in the fluorescence intensity and in the surface area of the FISH signal.
- 8-OHdG assay. One of the latest assays reported is the evaluation of the level of oxidized guanine residue that gives an indication as to the degree of sperm DNA oxidative damage. To date it is essentially used for research purpose and has yet to be developed for clinical routine testing. Sperm 8-OHdG evaluation is of interest because it is known to be associated with the level of de novo mutation in the germline [205].

The present current minimum standard is assessment of seminal plasma by volume, appearance and liquefaction of the ejaculate, and, for spermatozoa, measurement of concentration, motility and morphology [206]. The above mentioned techniques are rarely used in routine clinical evaluation because the tested spermatozoa are unsuitable for clinical purpose afterwards. However, for some of them they do allow the establishment of a DNA fragmentation rate, a useful marker in the prediction of fertility. Studies have shown that the chance of spontaneous conception starts to decline at sperm DNA damage (DFI) values above 20% and are close to zero for readings over 30–40% [207]. In another study using the Comet assay, the authors also showed that there was a strong correlation between sperm DNA fragmentation and fertility status of men [134]. Thus, there is robust evidence from all the DNA fragmentation assays that the chance of spontaneous pregnancy is reduced when sperm DNA damage is excessive.

## Conclusions

Thus, it appears that sperm defective nuclear condensation and sperm nuclear fragmentation are nowadays easy situations to assess with trustable/reliable direct and/or indirect assays (see above). Since Evenson et al. [208] highlighted the importance of sperm DNA damage evaluation our knowledge on the causes and consequences of the sperm DNA damage has increased, as well as the use of ART. In some countries, ART represents almost 5% of births, and the latest report indicates that to date approximately 5 million children were born from ART worldwide [209]. As part of the ART tool box, ICSI was introduced in 1992 and currently represents 60 to 80% of the cycles performed worldwide [210]. This is because the injection of a sperm into an oocyte bypasses all the selection processes that occurs in natural fertilization. This allows clinicians to cover more infertile situations especially those where the male partner is key due to low sperm numbers, morphologically abnormal sperm and hardly or no motile sperm. Because of the predominance of ICSI clinicians have to be very cautious as to the quality of the spermatozoa they select, especially concerning the integrity of the nuclear material. They also have to be cautious that by the selection protocols they use they do not further alter the sperm nuclear compartment. Despite the precautions taken in today sperm selection protocols, no one is able to tell that the spermatozoa chosen is free of nuclear alterations. Animal studies have shown that a morphologically normal sperm can present nuclear alterations that are detrimental to reproductive success. It is likely that this situation exists also in humans and that such spermatozoa can be selected for ICSI [211]. This issue is aggravated by the fact that it is difficult to separate the risk derived from the technique itself from the contribution of the integrity of the sperm nucleus to the offspring health after ART [212]. This renders animal models extremely important and valuable to evaluate more deeply the impact of the sperm nuclear alterations on the health of the progeny when ART is used. However, with the assays available today to evaluate the integrity of the sperm nucleus/DNA and consequently the quality of the paternal chromosomal material prior to ART, one could improve its safety and success rate. Even-though these assays will not solve the primary question of ICSI; whether the spermatozoa being used is in a perfect nuclear condition? they surely will be helpful in assessing the overall level of DNA damage existing in the sperm sample. It certainly will put ART clinicians in a challenging situation since what would be the answer to provide to a couple where the male is diagnosed with a very high level of DNA damage? Despite this difficulty, to our minds, the principle of caution should here prevail. Definitely, more research is needed to establish reliable thresholds that would lower the risk taken when using such DNA damaged spermatozoa. More research is also needed to investigate potential therapeutic actions that could improve the quality of the sperm nucleus. Simple therapeutic actions such as the correction of lifestyles and/or oral supplementations may prove useful in some situations [213, 214]. Some argue, why are we asking ART to do better than natural conception? We think that this is incorrect thinking, since we mainly would like ART (and especially ICSI) not to do worse. In natural conception, notwithstanding the female component, with all the existing barriers there are very few chances that a morphologically and functionally defective spermatozoa will fertilize an oocyte. With ICSI, a morphologically and functionally abnormal sperm cell may be allowed to fertilize. The integrity of the sperm nucleus being so tightly linked with the head morphology and in consequence the mobility of the cell it is very likely that an abnormal spermatozoa bears an abnormal nucleus. Therefore, monitoring the quality of the sperm nucleus by various means should be an integral part of the routine check-up of the male partner prior to ART.

## Abbreviations

3′-OH: 3′ hydroxyl
AO: Acridine orange
ART: Assisted reproductive technologies
BER: Base Excision Repair
CENP-A: Centromere-specific protein A
ChIP: Chromatin immunoprecipitation
CMA3: Chromomycin A3
DBD-FISH: DNA breakage detection fluorescence *in situ* hybridization
DFI: DNA fragmentation Index
DTT: Dithiotreitol
FISH: Fluorescence *in situ* hybridization
H_2_O_2_: Hydrogen peroxide
ICSI: IntraCytoplasmic Sperm Injection
IVF: In vitro fertilization
MAR: Matrix attachment regions
MSOME: Motile Sperm Organelle Morphology Examination
PARP: poly(ADP-ribose) [PAR] polymerases
Prm: Protamine
PTM: Post-translational modifications
PUFA: Polyunsaturated fatty acids
ROS: Reactive oxygen species
SCD: Sperm chromatin dispersion assay
SCSA: Sperm Chromatin Structure Assay
ssDNA: Single strand DNA
TEM: Transmitted electronic microscopy
TH2B: Testis-specific H2B variant
Tnp: Transition proteins
TUNEL: Terminal deoxynucleotidyl transferase-mediated deoxyrudine triophosphate Nick End Labeling
